# Optimized management strategy increased grain yield, promoted nitrogen balance, and improved water productivity in winter wheat

**DOI:** 10.3389/fpls.2023.1182568

**Published:** 2023-05-30

**Authors:** Haicheng Xu, Mei Liu, Yuhai Tang, Fei Zhao, Wenchao Cao, Mingrong He, Dianliang Peng, Xinglong Dai

**Affiliations:** ^1^ Shandong Facility Horticulture Bioengineering Research Center, Weifang University of Science and Technology, Weifang, China; ^2^ State Key Laboratory of Crop Biology, Agronomy College of Shandong Agricultural University, Tai’an, China

**Keywords:** optimized management strategy, grain yield, nitrogen balance, water productivity, winter wheat

## Abstract

The increasing costs of agricultural production and environmental concerns reinforce the need to reduce resource inputs. Improvements in nitrogen (N) use efficiency (NUE) and water productivity (WP) are critical for sustainable agriculture. We aimed to optimize management strategy to increase wheat grain yield, promote N balance, and improve NUE and WP. A 3-year experiment was conducted with four integrated treatments: conventional practice treatment (CP); improvement of conventional practice treatment (ICP); high-yield management treatment (HY), which aimed for maximizing grain yield regardless of resource inputs cost; and integrated soil and crop system management treatment (ISM), which aimed for testing an optimal combination of sowing date, seeding rate, and fertilization and irrigation management. The average grain yield for ISM was 95.86% of that for HY and was 5.99% and 21.72% higher than that for ICP and CP, respectively. ISM promoted N balance as relatively higher aboveground N uptake, lower inorganic N residue, and lowest inorganic N loss. The average NUE for ISM was 4.15% lower than that for ICP and was remarkably higher than that for HY and CP by 26.36% and 52.37%, respectively. The increased soil water consumption under ISM was mainly due to its increased root length density. Along with a high level of grain yield, ISM obtained a relatively adequate water supply due to the effective use of soil water storage, thereby increasing the average WP by 3.63%–38.10% in comparison with other integrated management treatments. These results demonstrated that optimized management strategy (appropriately delaying sowing date, increasing seeding rate, and optimizing fertilization and irrigation management) used under ISM could promote N balance and improve WP while increasing grain yield and NUE in winter wheat. Therefore, ISM can be considered a recommendable management strategy in the target region.

## 1 Introduction

In 2015, approximately 25.2 million tons of nitrogen (N) fertilizers were applied in China, where wheat production accounts for 13.5% of this N consumption (Heffer et al., 2017). Less than 50% of the N applied to farmland is recovered in crop plants (West et al., 2014). The excess N could be responsible for air pollution, soil acidification, water quality degradation, and increased greenhouse gas emission (Guo et al., 2010; Jiao, 2010; Zhang et al., 2015; Cui et al., 2018). The Huang-Huai-Hai Plain of China (HPC) is the major region of crop production in China, producing more than 70% of domestic wheat (National Bureau of Statistics of China, 2021). In the HPC, the water consumption of high-yielding winter wheat is about 450−650 mm (Huang et al., 2016). Nevertheless, rainfall during wheat-growing season can merely meet 25%−40% of crop water requirement (Zhang et al., 2006). That means additional irrigation is required. However, in farmer’s fields, excessive and irregular irrigation usually results in severe water waste and unsustainable grain yield (Guo et al., 2014; Man et al., 2015). Hence, optimized management strategies will be needed to improve wheat yield, N use efficiency (NUE), and water productivity (WP) while minimizing adverse environmental impacts.

Crop production alters N balance in aboveground N uptake (AGN), soil inorganic N residue (INR), and inorganic N loss (INL), ultimately affecting NUE and environmental health (Puntel et al., 2016; Liu et al., 2018; Li et al., 2022). The better N balance implies higher NUE and lower N loss. In the HPC, winter wheat–summer maize rotation is the predominant cropping system. In this system, poor uptake and utilization of residual soil nitrate N after harvest (Ju et al., 2006; Lu et al., 2021) leads to resource waste and environmental pollution. Numerous studies have shown that the proper management strategy can increase AGN or NUE, including optimized N management (Hartmann et al., 2015; Lu et al., 2015), proper irrigation (Wang et al., 2014; Ye et al., 2022), and appropriate seeding rate (Dai et al., 2013; Dai et al., 2014), thereby reducing INR and INL. In addition, the N nutrition index (NNI) can be used as an indicator of N uptake at canopy level (Lemaire et al., 2008). Agronomic practices such as fertilization and irrigation management can easily influence NNI (Lemaire and Gastal, 1997). Furthermore, many previous studies have demonstrated that the correlation between NUE and NNI was significantly negative (Dordas, 2011; Hu et al., 2014).

Root distribution directly affects crop nutrient uptake and soil water absorption. The cultivar, soil type, and fertilization and irrigation management practices can significantly affect the growth and spatial distribution of root systems (Dai et al., 2014; Wang et al., 2014; Man et al., 2016; Dai et al., 2017; Feng et al., 2023). A larger root system can commonly lead to the increases in total water consumption through the increases in extraction of the stored soil water (Cooper et al., 1987). The increased water absorption from the deep soil may be helpful for wheat canopy to maintain grain production (Walter and Moril, 2005). Therefore, optimizing the vertical distribution of wheat root system is important to improve the grain yield and WP.

Wheat production is influenced by a combination of various cultivation factors. To determine the effects of combined cultivation factors is more representative for wheat production studies. The integrated management strategy is defined as a comprehensive management framework including appropriate variety, sowing date, seeding rate, and fertilization and irrigation management (Chen et al., 2011; Chen et al., 2014; Cui et al., 2018). The better performance in terms of grain yield and resource efficiency has been achieved (Xu H. C. et al., 2018; Cheng et al., 2020). In an earlier study, we have elucidated the differences in wheat grain yield formation and N use under four integrated management treatments (Xu H. C. et al., 2018). However, the effects of integrated management practices on N balance and water utilization are poorly understood.

Therefore, we wonder whether the optimized management strategy could promote N balance and improve WP while increasing grain yield and NUE in winter wheat. We evaluated the effects of integrated management strategy on crop N accumulation and redistribution, crop N status, direction and proportion of soil inorganic N, soil water consumption, and WP. The objective of the present study was to determine whether the optimized management strategy could increase grain yield, promote N balance, and improve NUE and WP in the target region.

## 2 Materials and methods

### 2.1 Site description

**Figure 1:**
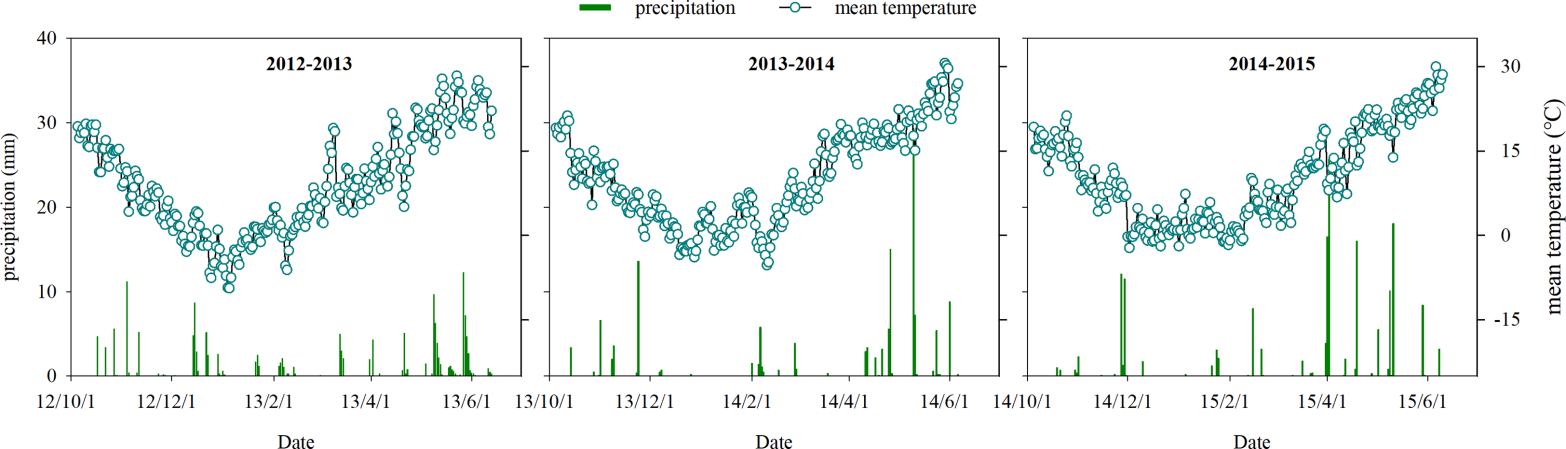
The mean temperature and precipitation recorded during the 2012 to 2015 wheat-growing seasons in experimental fields.

As a part of a long-term field experiment with wheat starting in 2009, field experiments were conducted from 2012 to 2015 in Tai’an, Shandong Province, China (35°58′N, 117°03′E), which experiences a temperate continental monsoon climate. The annual cumulative temperature above 10°C and solar duration were at least 4,213°C and 2,627 h, respectively. The frost-free period in 1 year was 195 days. The annual average precipitation and rainfall during wheat grown season between 1981 and 2012 were 754.34 and 193.63 mm. The soil was sandy loam. Organic matter, total N, available phosphorous (P), and available potassium (K) in the upper 0.2 m of the soil before sowing in 2012−2013, 2013−2014, and 2014−2015 are shown in **Table 1**. The winter wheat–summer maize rotation was performed in this area. The previous summer maize straw was returned to the soil every year. Precipitation during the three wheat-growing seasons was 157.20, 132.40, and 164.30 mm, respectively (**Figure 1**). All meteorological data were collected from an automatic weather station (CR800 data logger, Campbell Scientific, Rockford, IL, USA).

**Table 1:** Nutrient status of top 0- to 20-cm soil before sowing in 2012−2013, 2013−2014, and 2014−2015 growing seasons.

Year	Organic matter (g kg^−1^)	Total nitrogen (g kg^−1^)	Available phosphorous (mg kg^−1^)	Available potassium (mg kg^−1^)
2012−2013	16.72	1.05	22.65	86.15
2013−2014	16.86	1.08	27.35	89.95
2014−2015	16.90	1.12	27.10	85.52

### 2.2 Experimental design

The following four integrated management treatments were included in a randomized complete block design with four replicates: CP (conventional practice treatment), ICP (improvement of conventional practice treatment), HY (high-yield management treatment), and ISM (integrated soil and crop system management treatment) were carried out. For CP, the conventional planting practices of the local farmers were conducted; for ICP, it was based on improving conventional farmers’ practices in the region, mostly through increasing seeding rate, delaying sowing date, and decreasing fertilizer rate and irrigation amount, to increase wheat yield and improve resource efficiency; for HY, the purpose was to test wheat yield potential by adjusting the management practices such as further increased seeding rate, further increased fertilizer rate, and revised fertilization timing, regardless of the resource inputs cost; for ISM, the whole wheat production system based on the local environment was redesigned, drawing on appropriate delayed sowing date, a higher seeding rate, and lower fertilizer or irrigation input compared with HY. The size of each experimental plot was 120 m^2^ (3 × 40 m) with 12 rows (0.25 m apart). The widely planted winter wheat variety “Tainong 18”, which was the cultivar with large ears and low tillering capacity, was grown in the field.

The combination details of sowing date, seeding rate, and fertilization and irrigation management are shown in **Table 2**. Sowing dates for the four integrated treatments were 5, 8, 8, and 12 October, respectively. Seeding rates increased by 75 seeds m^−2^ with each integrated treatment from CP (225 seeds m^−2^) to ISM (450 seeds m^−2^). Urea, calcium superphosphate, and potassium chloride were used as the N, P, and K fertilizers. For each treatment, 210−315 kg N ha^−1^, 90−210 kg P_2_O_5_ ha^−1^, and 30−150 kg K_2_O ha^−1^ were applied. N topdressing for CP was applied at the regreening stage, whereas that for ICP, HY, and ISM was applied at the jointing stage. The basal and topdressing ratios of N fertilizer for four integrated treatments were 6:4, 5:5, 4:6, and 4:6, respectively. P fertilizers were applied as basal fertilizers prior to wheat drilling for all integrated treatments. K fertilizers for both CP and ICP were applied as basal fertilizers. K topdressing for HY and ISM were applied at jointing stage, and the basal and topdressing ratios of K fertilizer for both HY and ISM were 6:4. Over 3 years, CP was irrigated five times (post-sowing, before winter, regreening, anthesis, and anthesis + 15 days), and ICP and HY were irrigated four times (post-sowing, jointing, anthesis, and anthesis + 15 days). ISM was irrigated four times (post-sowing, jointing, anthesis, and anthesis + 15 days) in 2012−2013 and 2013−2014 and was irrigated three times (post-sowing, jointing, and anthesis) in 2014−2015. The amount of irrigation was 70 mm each time, which was measured by a water meter. No significant incidences of weeds, pests, or diseases were observed in each plot. In addition, treatment plots with no applied N were established. All conditions were the same as described above, except for N application.

**Table 2:** The seeding rate, sowing date, nutrients, and irrigation management used in the different integrated treatments.

Treatment^1)^	Seeding rate (seeds m^−2^)	Sowing date (m/d)	The periods and rates of fertilizer application (kg ha^−1^)				The periods and rates of irrigation (mm)					
			Fertilizer	Pre seeding	Regreening	Jointing	Post-sowing	Before winter	Regreening	Jointing	Anthesis	Anthesis + 15 days
CP	225	10/5	N	189	126	−	70	70	70	−	70	70
			P	120	−	−						
			K	30	−	−						
ICP	300	10/8	N	105	−	105	70	−	−	70	70	70
			P	90	−	−						
			K	75	−	−						
HY	375	10/8	N	126	−	189	70	−	−	70	70	70
			P	210	−	−						
			K	90	−	60						
ISM	450	10/12	N	96	−	144	70	−	−	70	70	or 70
			P	120	−	−						
			K	45	−	30						

^1)^ CP, conventional practice treatment; ICP, improvement of conventional practice treatment; HY, high-yield management treatment; ISM, integrated soil and crop system management treatment. –, no data.

### 2.3 Sampling and measurements

#### 2.3.1 N accumulation and transport

The representative plants from a 1.0-m^2^ quadrat in each plot were collected at anthesis and maturity. Samples were divided into leaves, stems + sheaths, spikes (ear rachises + glumes), and grains (only at maturity). The wheat plant components were dried at 105°C for 15 min, oven-dried at 70°C until constant weight, and subsequently weighed. N concentration was determined using the Kjeldahl method (Bremner and Tabatabai, 1972). N accumulation of each wheat plant component was calculated by obtaining the product of dry matter weight and N concentration. The AGN (kg ha^−1^) was calculated as the sum of N accumulation of the measured wheat plant components. NNI was estimated according to the ratio of the actual plant N concentration and the critical N concentration at anthesis, where the latter was estimated according to the dilution curve for winter wheat described by Justes et al. (1994). Post-anthesis N remobilization (NR; kg ha^−1^), post-anthesis N uptake (PANU; kg ha^−1^), NR efficiency (NRE; %), and contribution proportion of NR and PANU to N accumulation in grains (N-grain; kg ha^−1^) were calculated as described by Moll et al. (1982) and Gaju et al. (2014), as follows:


$$\text{NR}={\text{N}}_{\text{A}}-{\text{N}}_{\text{M}}$$

$$\text{NRE}=\left({\text{N}}_{\text{A}}-{\text{N}}_{\text{M}}\right)/{\text{N}}_{\text{A}}$$

$$\text{PANU}={\text{AGN}}_{\text{M}}-{\text{AGN}}_{\text{A}}$$

$${\text{CP}}_{\text{NR}}=\text{NR}/\text{N}-\text{grain}$$

$${\text{CP}}_{\text{PANU}}=\text{PANU}/\text{N}-\text{grain}$$

where N_A_ is the N accumulation in the crop component at anthesis (kg ha^−1^), N_M_ is the N accumulation in the crop component at maturity (kg ha^−1^), AGN_M_ is the AGN at maturity (kg ha^−1^), AGN_A_ is the AGN at anthesis (kg ha^−1^), CP_NR_ is the contribution proportion of NR to N-grain (%), and CP_PANU_ is the contribution proportion of PANU to N-grain (%).

#### 2.3.2 Soil inorganic N

Soil samples were collected from each treatment plot at random with 0.2-m increments up to a depth of 1 m prior to wheat drilling and at maturity. Following the method described by Liu et al. (2003), the fresh soil samples collected from the same depth in each treatment plot were pooled and extracted immediately with KCl solution (1 mol L^−1^) to determine inorganic N (nitrate N and ammonium N) concentrations by using a continuous flow analyzer (Bran + Lubbe, Norderstedt, Germany). Available N was calculated as the sum of fertilizer N and INR in 0- to 100-cm-depth soil before sowing.

#### 2.3.3 N balance

The apparent N mineralization (ANM; kg ha^−1^) was calculated as the difference between the N output and N input in the plots without N fertilizer application (Olfs et al., 2005), as follows:


$$\text{ANM}={\text{AGN}}_{\text{M}}+{\text{INR}}_{\text{M}}-{\text{INR}}_{\text{S}}$$

The INL (kg ha^−1^) was calculated as the difference between the N input and the N output in the plots where N was applied (Yin et al., 2018), as follows:


$$\text{INL}={\text{INR}}_{\text{S}}+\text{ANM}+\text{NFA}-{\text{AGN}}_{\text{M}}-{\text{INR}}_{\text{M}}$$

where INR_M_ is the INR at maturity (kg ha^−1^), INR_S_ is the INR before sowing (kg ha^−1^), NFA is the N fertilizer amount (kg ha^−1^).

#### 2.3.4 Soil water consumption and total water consumption

Soil moisture was measured with 0.2-m increments up to a depth of 2 m in each plot by using a time domain reflectometry (Trime-Pico, Germany). The soil water consumption in the 0- to 2-m-depth soil was calculated as the difference between the soil water content before sowing and at maturity. At the study site, groundwater runoff and recharge can be ignored. Total water consumption was calculated as the sum of seasonal precipitation, irrigation amount, and soil water consumption (Li et al., 2019).

#### 2.3.5 Root length

Roots measurements were made only in 2014−2015. Root sampling was conducted at anthesis stage. In each integrated treatment, we sampled roots from a representative area of the site occupied by a group of wheat plants. The area was 0.4 m wide (measured parallel to the rows) and 0.5 m long (measured perpendicular to the rows and covering wheat plants in two rows). Each cubic soil sample was divided into 0.2-m increments to a depth of 1.4 m. All root samples were sorted by washing out any soil and dyed immediately with methyl blue for 12 h. The root samples were scanned with a flatbed scanner (HP Scanjet 8200, Hewlett–Packard, Palo Alto, CA, USA) and were analyzed with an image analyzer (Delta-T Devices Ltd, Cambridge, UK) to measure the root lengths, consistent with Dai et al. (2013; 2014). Root length density (RLD; mm cm^−3^) was calculated as the total length of roots divided by the corresponding soil volume. The depths above which 95% of the roots were located (D_95_) were calculated to summarize the vertical distribution of RLD (Hund et al., 2009) and were calculated as described by Dai et al. (2014).

#### 2.3.6 Grain yield, N utilization, and water productivity

Grain yield was determined from a 3-m^2^ quadrat (with six rows, 2 m apart) in each experimental plot at maturity. The wheat grain was naturally air-dried, weighed, and then adjusted to the standard 13% moisture content. NUE (kg kg^−1^) was defined as the grain yield per unit available N (Moll et al., 1982). N fertilizer productivity (NFP; kg kg^−1^) was defined as the grain yield per unit fertilizer N. WP (kg ha^−1^ mm^−1^) was calculated by dividing the grain yield by total water consumption (Xu X. X. et al., 2018).

### 2.4 Statistical analysis

Statistical analysis was performed with DPS version 7.05 software (Hangzhou Ruifeng Information Technology Co., Ltd., China). Multiple comparisons were performed after a preliminary F-test. Differences between means were evaluated on the basis of the least significant difference at P< 0.05. Drawing figures were conducted by using SigmaPlot 12.5 (Systat Software, San Jose, CA, USA).

## 3 Results

### 3.1 N accumulation and partitioning at anthesis

Year and treatment significantly affected AGN_A_, whereas their interaction did not. Over 3 years, AGN_A_ among the integrated treatments followed the ranking HY > ISM > ICP ≈ CP (**Table 3**). HY consistently achieved the highest AGN_A_. No remarkable difference in AGN_A_ was observed between CP and ICP. The average AGN_A_ for ISM was 84.73% of that for HY and was 12.07% higher than that for CP.

**Table 3:** N accumulation in crop components, aboveground N uptake (AGN_A_), and N nutrition index (NNI_A_) at anthesis under different integrated treatments from 2012 to 2015.

Year	Treatment^1)^	AGN_A_ (kg ha^−1^)	N accumulation (kg ha^−1^)			NNI_A_
			Stem	Leaf	Spike	
2012−2013	CP	199.54 c	77.88 d	76.65 c	45.01 d	1.03 d
	ICP	203.39 c	79.97 c	77.11 c	46.31 c	1.05 c
	HY	269.42 a	100.16 a	113.26 a	56.00 a	1.29 a
	ISM	221.98 b	84.01 b	89.73 b	48.24 b	1.12 b
2013−2014	CP	202.56 c	82.43 d	78.43 c	41.70 bc	1.06 d
	ICP	207.55 c	90.24 c	76.80 c	40.52 c	1.08 c
	HY	268.15 a	114.38 a	104.74 a	49.03 a	1.31 a
	ISM	232.23 b	101.50 b	87.24 b	43.49 b	1.18 b
2014−2015	CP	230.24 c	86.10 d	99.76 c	44.38 c	1.11 d
	ICP	237.32 c	90.87 c	103.60 bc	42.85 c	1.15 c
	HY	298.86 a	110.75 a	131.53 a	56.58 a	1.36 a
	ISM	254.46 b	97.07 b	106.90 b	50.50 b	1.21 b
Mean	CP	210.78 c	82.14 c	84.95 c	43.70 c	1.07 c
	ICP	216.09 c	87.03 c	85.84 bc	43.22 c	1.09 c
	HY	278.81 a	108.43 a	116.51 a	53.87 a	1.32 a
	ISM	236.22 b	94.19 b	94.62 b	47.41 b	1.17 b
*P*-value						
Year (Y)		0.0001	0.0001	0.0001	0.0001	0.0001
Treatment (T)		0.0001	0.0001	0.0001	0.0001	0.0001
Y×T		0.1193	0.0001	0.0066	0.0041	0.0001

^1)^ CP, conventional practice treatment; ICP, improvement of conventional practice treatment; HY, high-yield management treatment; ISM, integrated soil and crop system management treatment. Values followed by different letters within a column in the same year are significantly different at P< 0.05.

The effects of treatment, year, and their interaction on N accumulation in crop components and NNI at anthesis (NNI_A_) were significant (**Table 3**). Over 3 years, the stem N among the integrated treatments had the following rank order: HY > ISM > ICP > CP. For the leaf N, the order was HY > ISM ≥ ICP ≈ CP. For the spike N, the order was HY > ISM ≥ ICP ≥ CP. The N accumulation in all crop components was the highest for HY, followed by ISM, and the lowest for CP. NNI_A_ was used as an indicator of N uptake at canopy level incurred by the integrated treatments. Over 3 years, NNI_A_ among all integrated treatments followed the ranking HY > ISM > ICP > CP. The values of NNI were all more than 1, which indicated that wheat grown under all the integrated treatments was in situation of a luxury N consumption.

### 3.2 N remobilization and post-anthesis N uptake

Year, treatment, and their interaction significantly affected N accumulation in grains (N-grain) at maturity. N-grain among the integrated treatments followed the ranking HY > ISM > ICP > CP. The average N-grain for ISM was 91.80% of that for HY and was 9.63% and 26.43% higher than that for ICP and CP, respectively (**Table 4**).

**Table 4:** N accumulation in grains (N-grain), N remobilization (NR), N remobilization efficiency (NRE), post-anthesis N uptake (PANU), contribution proportion of NR to N-grain (CP_NR_), and contribution proportion of PANU to N-grain (CP_PANU_) under different integrated treatments from 2012 to 2015.

Year	Treatment^1)^	N-grain (kg ha^−1^)	NR (kg ha^−1^)	NRE (%)	CP_NR_ (%)	PANU (kg ha^−1^)	CP_PANU_ (%)
2012−2013	CP	161.52 d	142.71 d	71.52 b	88.36 a	18.81 c	11.64 b
	ICP	183.07 c	151.92 c	74.69 a	83.00 b	31.15 b	17.00 a
	HY	215.14 a	194.43 a	72.17 b	90.39 a	20.71 c	9.61 b
	ISM	200.42 b	164.60 b	74.15 a	82.14 b	35.82 a	17.86 a
2013−2014	CP	159.85 d	144.83 d	71.50 b	90.62 a	15.02 c	9.38 c
	ICP	186.04 c	151.95 c	73.21 a	81.69 c	34.09 b	18.31 a
	HY	224.21 a	191.61 a	71.45 b	85.48 b	32.60 b	14.52 b
	ISM	207.00 b	167.69 b	72.21 ab	81.02 c	39.31 a	18.98 a
2014−2015	CP	173.23 d	162.86 d	70.74 b	94.02 a	10.36 c	5.98 c
	ICP	200.48 c	174.06 c	73.34 a	86.83 b	26.41 b	13.17 b
	HY	240.02 a	211.28 a	70.70 b	88.04 b	28.74 b	11.96 b
	ISM	217.43 b	184.55 b	72.53 a	84.88 c	32.88 a	15.12 a
Mean	CP	165.64 d	151.48 d	71.20 c	91.38 a	14.17 c	8.62 c
	ICP	191.02 c	160.79 c	73.66 a	84.09 c	30.23 b	15.91 a
	HY	228.11 a	200.23 a	71.34 c	87.81 b	27.89 b	12.19 b
	ISM	209.42 b	173.61 b	72.85 b	82.86 c	35.81 a	17.14 a
*P*-value							
Year (Y)		0.0001	0.0001	0.0001	0.0001	0.0001	0.0001
Treatment (T)		0.0001	0.0001	0.0001	0.0001	0.0001	0.0001
Y×T		0.0001	0.0004	0.2209	0.0001	0.0001	0.0001

^1)^ CP, conventional practice treatment; ICP, improvement of conventional practice treatment; HY, high-yield management treatment; ISM, integrated soil and crop system management treatment. Values followed by different letters within a column in the same year are significantly different at P< 0.05.

The effects of treatment, year, and their interaction on post-anthesis NR were significant (**Table 4**). Over 3 years, NR among the integrated treatments exhibited the same rank order as N-grain. The average NR for ISM was 86.71% of that for HY and was 7.98% and 14.61% higher than that for ICP and CP, respectively. Year and treatment significantly affected NRE, whereas their interaction did not. Over 3 years, the NRE was the largest for ICP and ISM and the smallest for CP and HY. No remarkable differences in NRE were observed between ICP and ISM and between CP and HY. The average NRE for ISM was 2.12% and 2.32% higher than that for HY and CP, respectively. Year, treatment, and their interaction significantly affected the contribution proportion of NR to N-grain (CP_NR_). The CP_NR_ was the largest for CP and the smallest for ISM. The CP_NR_ obtained with HY in 2012−2013 and 2013−2014, respectively, was significantly higher than that with ICP, whereas no remarkable difference in CP_NR_ was observed between ICP and HY in 2014−2015. The effects of treatment, year, and their interaction on PANU and the contribution proportion of PANU to N-grain (CP_PANU_) were significant (**Table 4**). PANU for the integrated treatments followed a similar ranking of ISM > ICP ≈ HY > CP in 2013−2014 and 2014−2015 except in 2012−2013, where the ranking of treatments was ISM > ICP > HY ≈ CP. The average PANU across 3 years for ISM was significantly higher than that for ICP, HY, and CP by 18.43%, 28.40%, and 152.76%, respectively. The CP_PANU_ was the largest for ISM and the smallest for CP. The CP_PANU_ obtained with ICP in 2012−2013 and 2013−2014, respectively, was significantly higher than that with HY, whereas no remarkable difference in CP_PANU_ was observed between ICP and HY in 2014−2015. The average CP_PANU_ for ISM was 7.75%, 40.59%, and 98.75% higher than that for ICP, HY, and CP, respectively. These results showed that the highest NR was the main reason HY produced the highest N-grain. ISM increased N-grain mainly due to the increases in both NR and PANU and the improvement of NRE compared with CP and ICP.

### 3.3 N balance

The effects of treatment, year, and their interaction on ANM and INR_S_ were significant (**Table 5**). The ANM among all integrated treatments had the following rank order: HY > ISM > ICP ≥ CP. Over 3 years, the INR_S_ was the highest for HY and the lowest for ICP. The INR_S_ obtained with ISM in 2012−2013 was significantly higher than that with ICP, whereas no remarkable differences in INR_S_ were observed between ISM and ICP in 2013−2014 and 2014−2015. The average INR_S_ for ISM was 23.24% and 14.27% lower than that for HY and CP, respectively.

**Table 5:** Apparent N mineralization (ANM), N fertilizer amount (NFA), inorganic N residue before sowing (INR_S_), aboveground N uptake at maturity (AGN_M_), inorganic N residue at maturity (INR_M_), and inorganic N loss (INL) under different integrated treatments from 2012 to 2015.

Year	Treatment^1)^	ANM (kg ha^−1^)	NFA (kg ha^−1^)	INR_S_ (kg ha^−1^)	AGN_M_ (kg ha^−1^)	INR_M_ (kg ha^−1^)	INL (kg ha^−1^)
2012−2013	CP	57.17 d	315.00	252.24 a	218.35 d	194.52 b	211.54 a
	ICP	64.05 c	210.00	168.99 c	234.54 c	120.02 d	88.49 d
	HY	82.70 a	315.00	253.61 a	290.13 a	207.38 a	153.80 b
	ISM	72.26 b	240.00	200.00 b	257.80 b	154.00 c	100.47 c
2013−2014	CP	54.74 c	315.00	214.40 b	217.58 d	201.65 b	164.91 a
	ICP	55.95 c	210.00	189.57 c	241.64 c	126.35 d	87.53 d
	HY	87.03 a	315.00	255.32 a	300.75 a	208.46 a	148.15 b
	ISM	76.49 b	240.00	207.28 bc	271.53 b	157.29 c	94.95 c
2014−2015	CP	54.01 c	315.00	201.38 b	240.60 d	186.36 a	143.43 a
	ICP	56.44 c	210.00	170.69 c	263.73 c	97.54 c	75.85 c
	HY	79.84 a	315.00	237.10 a	327.60 a	185.23 a	119.11 b
	ISM	72.11 b	240.00	165.40 c	287.34 b	110.13 b	80.04 c
Mean	CP	55.30 d	315.00	222.67 b	225.51 d	194.18 a	173.29 a
	ICP	58.81 c	210.00	176.41 c	246.64 c	114.63 c	83.96 c
	HY	83.19 a	315.00	248.68 a	306.16 a	200.36 a	140.35 b
	ISM	73.62 b	240.00	190.89 c	272.22 b	140.47 b	91.82 c
*P*-value							
Year (Y)		0.0001	−	0.0001	0.0001	0.0001	0.0001
Treatment (T)		0.0001	−	0.0001	0.0001	0.0001	0.0001
Y×T		0.0001	−	0.0026	0.0001	0.0001	0.0001

^1)^ CP, conventional practice treatment; ICP, improvement of conventional practice treatment; HY, high-yield management treatment; ISM, integrated soil and crop system management treatment. Values followed by different letters within a column in the same year are significantly different at P< 0.05. –, no data.

**Figure 2:**
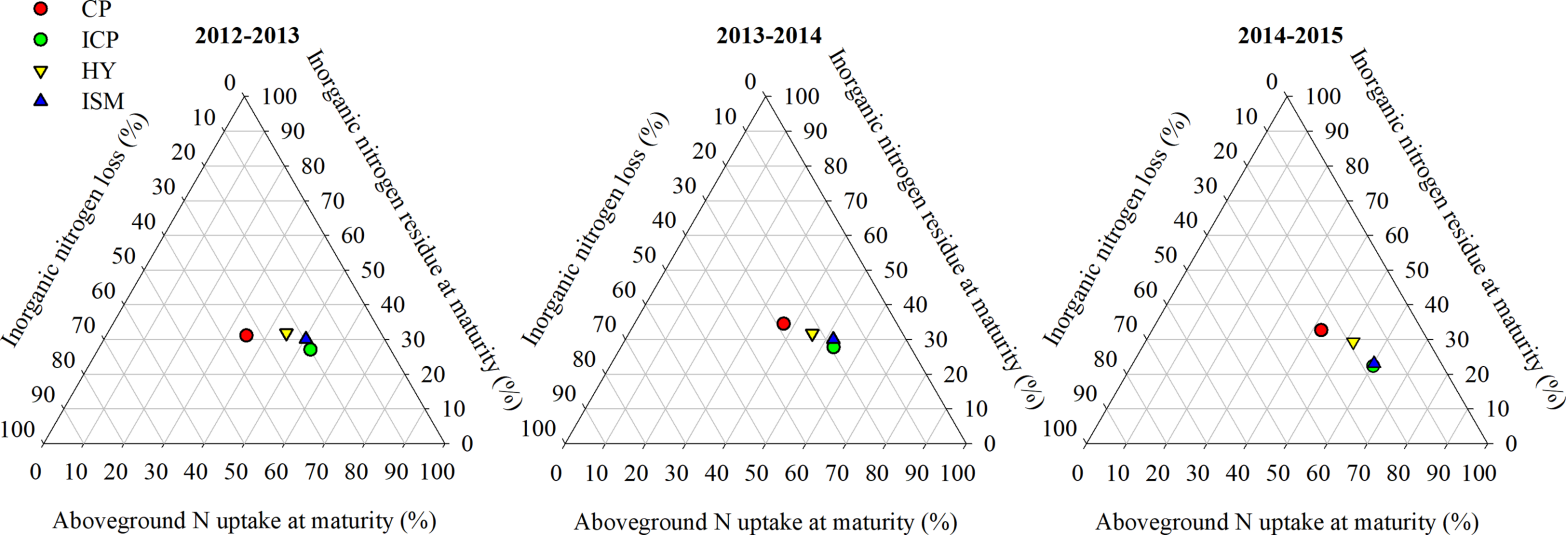
Direction and proportion of soil inorganic N under different integrated treatments from 2012 to 2015. CP, conventional practice treatment; ICP, improvement of conventional practice treatment; HY, high-yield management treatment; ISM, integrated soil and crop system management treatment.

The direction of soil inorganic N can be divided into three parts: AGN_M_, INR_M_, and INL (**Figure 2** and **Table 5**). Significant effects of treatment, year, and their interaction on AGN_M_, INR_M_, and INL were observed. Over 3 years, AGN_M_ among the integrated treatments followed the ranking HY > ISM > ICP > CP. The average AGN_M_ for ISM was 88.92% of that for HY and was 10.37% and 20.71% higher than that for ICP and CP, respectively. INR_M_ among all integrated treatments had the following rank order: HY ≥ CP > ISM > ICP. INR_M_ obtained with HY in 2012−2013 and 2013−2014 was significantly higher than that with CP, whereas no remarkable difference in INR_M_ was observed between HY and CP in 2014−2015. The average INR_M_ for ISM was 29.89% and 27.66% lower than that for HY and CP, respectively. INL among the integrated treatments followed the ranking CP > HY > ISM ≥ ICP. The INL obtained with ISM in 2012−2013 and 2013−2014, respectively, was significantly higher than that with ICP, whereas no remarkable difference in INL was observed between ISM and ICP in 2014−2015. The average INL for ISM was significantly lower than that for HY and CP by 34.58% and 47.01%, respectively. These indicated that the integrated management strategy used under ISM supported better N balance by producing a high level of AGN_M_ and decreasing INR_M_ and INL compared with HY and CP.

### 3.4 Total water consumption and soil water consumption

**Figure 3:**
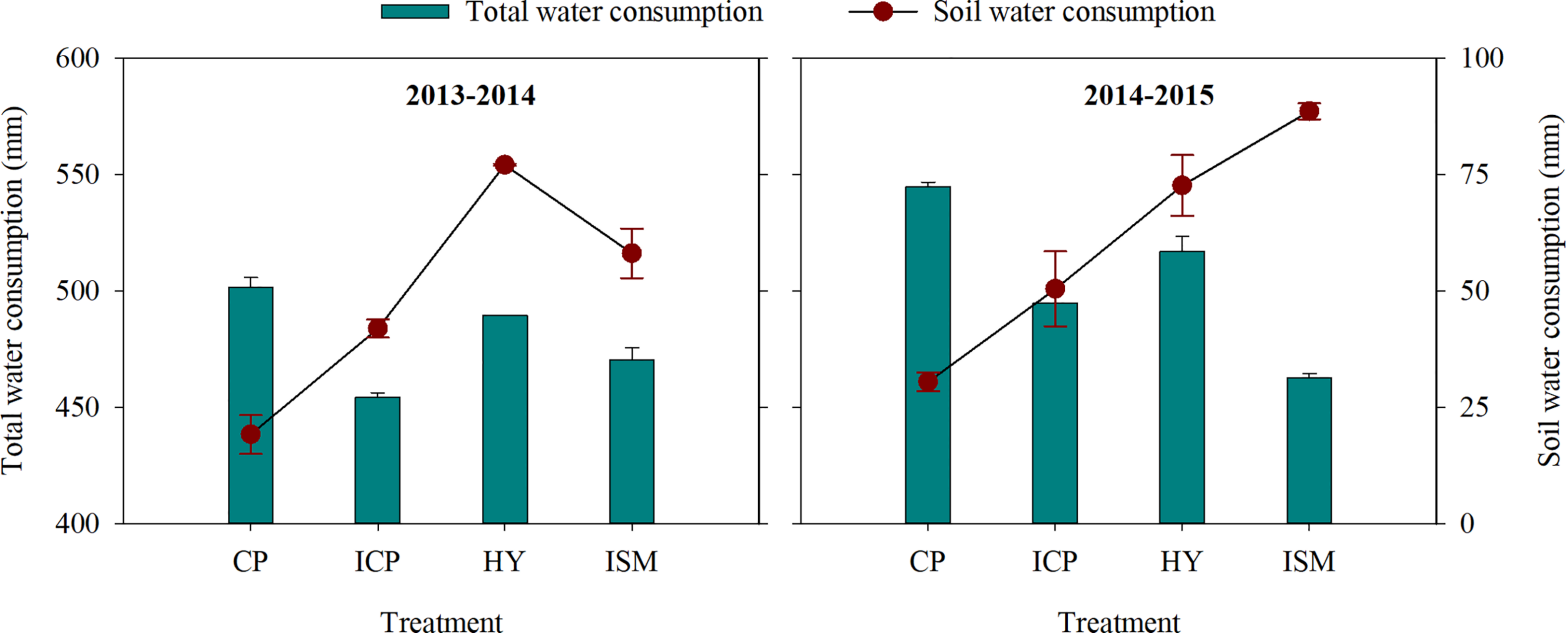
Total water consumption and soil water consumption during the entire growing season under different integrated treatments in 2013−2014 and 2014−2015. CP, conventional practice treatment; ICP, improvement of conventional practice treatment; HY, high-yield management treatment; ISM, integrated soil and crop system management treatment. Error bars represent the standard deviation of the mean.

**Figure 4:**
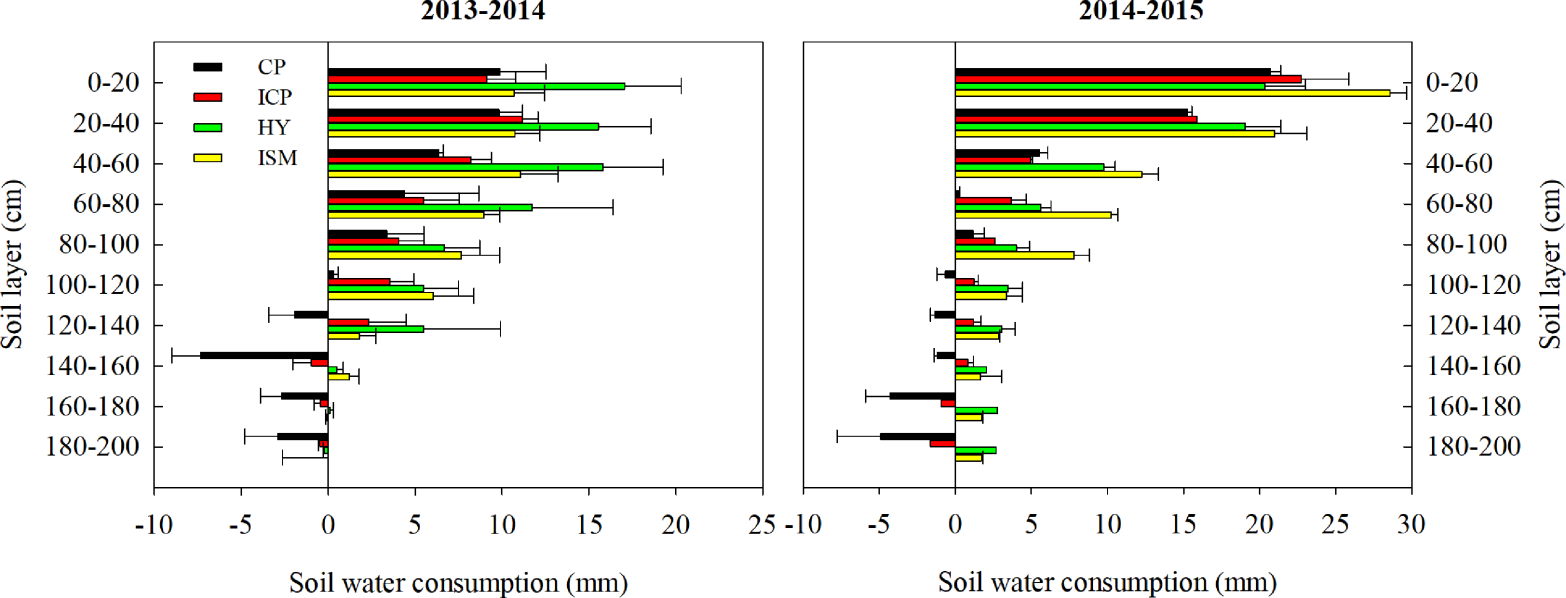
Soil water consumption at each 20-cm interval of soil depth in 0- to 200-cm soil layer during the entire growing season under different integrated treatments in 2013−2014 and 2014−2015. CP, conventional practice treatment; ICP, improvement of conventional practice treatment; HY, high-yield management treatment; ISM, integrated soil and crop system management treatment. Error bars represent the standard deviation of the mean.

Integrated treatments had a significant effect on total water consumption and soil water consumption in 0- to 200-cm-depth soil (**Figure 3**). In 2013−2014, total water consumption in ISM was 3.56% higher than that in ICP and was 3.88% and 6.20% lower than that in HY and CP, respectively. The soil water consumption in ISM was 24.64% lower than that in HY but was 38.51% and 202.26% higher than that in ICP and CP, respectively. In 2014−2015, the total water consumption in ISM was significantly lower than that in ICP, HY, and CP by 6.44%, 10.46%, and 15.03%, respectively. However, the soil water consumption in ISM was 21.89%, 75.55%, and 190.32% higher than that in HY, ICP, and CP, respectively. Over 2 years, the soil water consumption in the 0- to 200-cm soil layer obtained with ISM and HY was consistently higher than that with ICP and CP (**Figure 4**). The lowest soil water consumption in each soil layer was obtained with CP. We observed no significant differences in soil water consumption in the 100- to 200-cm soil layer between ISM and HY. However, inconsistent trends between HY and ISM over 2 years were observed. The soil water consumption in the 0- to 80-cm soil layer for ISM was lower than that for HY in 2013−2014 but was significantly higher than that for HY in 2014−2015. Compared with HY, the soil water consumption for ISM was remarkably higher in the 60- to 100-cm soil layer in 2014−2015. That is, ISM significantly increased the absorption of deep soil water.

### 3.5 Root distribution

**Figure 5:**
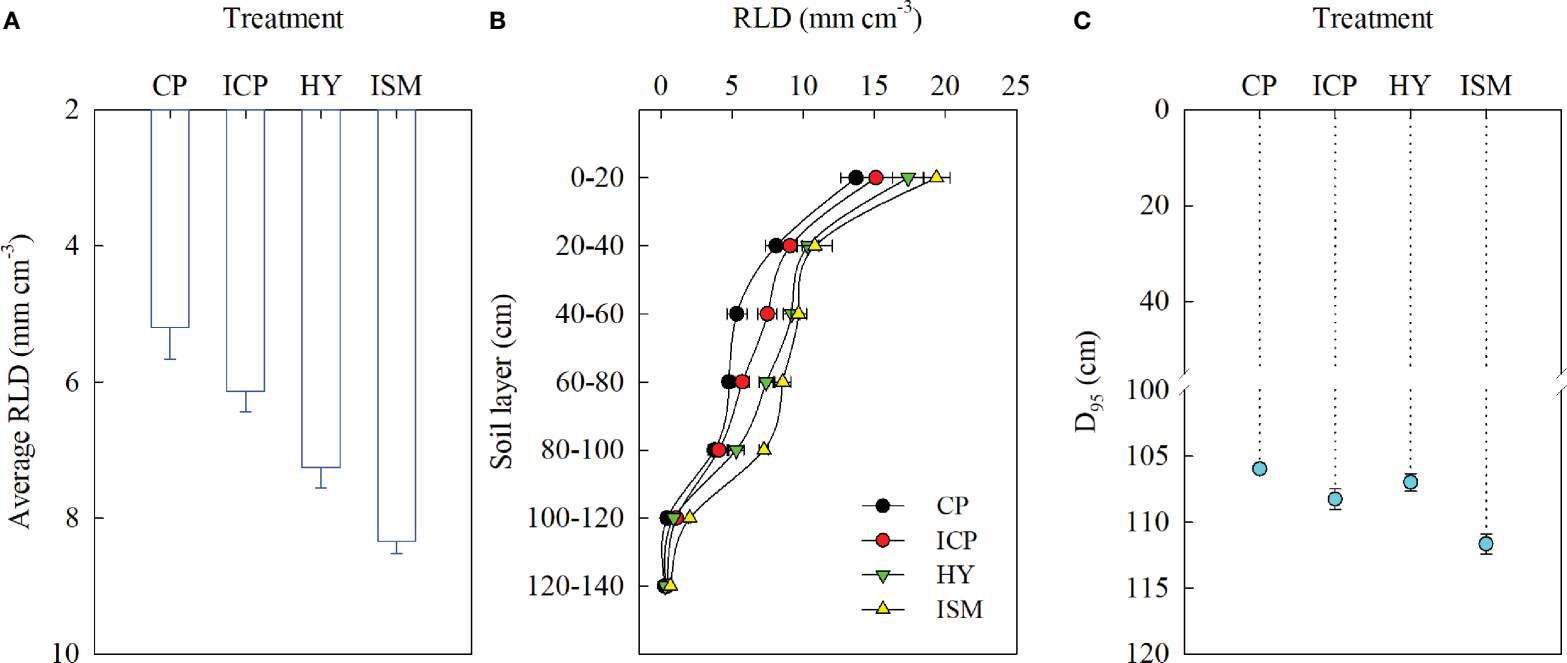
Average root length density (RLD) in 0- to 140-cm-depth soil **(A)**, RLD at each 20-cm interval of soil depth **(B)**, the depth above which 95% of the roots were located (D_95_) **(C)** under different integrated treatments in 2014−2015. CP, conventional practice treatment; ICP, improvement of conventional practice treatment; HY, high-yield management treatment; ISM, integrated soil and crop system management treatment. Error bars represent the standard deviation of the mean.

The root distribution in 0- to 140-cm-depth soil was significantly affected by integrated management practices. The average RLD obtained with ISM was 14.86%, 35.83%, and 60.45% larger than that with HY, ICP, and CP, respectively (**Figure 5A**). The RLD decreased with depth under all integrated treatments (**Figure 5B**). The RLD at each 20-cm interval of soil depth was the largest for ISM and the smallest for CP. The RLD in the 80- to 140-cm soil layer for ISM was significantly larger than that for HY, whereas no significant differences in RLD in the 0- to 80-cm soil layer were observed between ISM and HY. The RLD in the 40- to 60-cm soil layer for ICP was larger than that for CP, whereas no remarkable differences in other soil layers were observed between ICP and CP. The D_95_ among the integrated treatments followed the ranking ISM > ICP ≥ HY ≥ CP (**Figure 5C**). In summary, the integrated management strategy used under ISM could achieve the larger and deeper RLD than other integrated management treatments.

### 3.6 Grain yield, N utilization, and water productivity

Year and treatment significantly affected grain yield, but their interaction did not. Over 3 years, grain yield among all integrated treatments followed the ranking HY > ISM > ICP > CP. The average grain yield for ISM was 95.86% of that for HY and was 5.99% and 21.72% higher than that for ICP and CP, respectively (**Table 6**).

**Table 6:** Grain yield, N use efficiency (NUE), N fertilizer productivity (NFP), and water productivity (WP) under different integrated treatments from 2012 to 2015.

Year	Treatment^1)^	Grain yield (kg ha^−1^)	NUE (kg kg^−1^)	NFP (kg kg^−1^)	WP (kg ha^−1^ mm^−1^)
2012−2013	CP	7,188.16 d	12.67 d	22.82 d	−
	ICP	8,096.76 c	21.36 a	38.56 a	−
	HY	8,956.42 a	15.75 c	28.43 c	−
	ISM	8,672.35 b	19.71 b	36.13 b	−
2013−2014	CP	7,311.85 d	13.81 d	23.21 d	14.58 c
	ICP	8,476.34 c	21.21 a	40.36 a	18.66 b
	HY	9,466.16 a	16.60 c	30.05 c	19.34 a
	ISM	8,979.92 b	20.08 b	37.42 b	19.09 a
2014−2015	CP	7,417.84 d	14.37 d	23.55 d	13.62 d
	ICP	8,571.12 c	22.51 a	40.81 a	17.32 c
	HY	9,411.30 a	17.05 c	29.88 c	18.20 b
	ISM	9,026.53 b	22.27 b	37.61 b	19.40 a
Mean	CP	7,321.94 d	13.72 d	23.24 d	14.00 d
	ICP	8,408.51 c	21.81 a	40.04 a	17.86 c
	HY	9,297.01 a	16.55 c	29.51 c	18.66 b
	ISM	8,912.02 b	20.91 b	37.13 b	19.33 a
*P*-value					
Year (Y)		0.0001	0.0001	0.0001	0.0001
Treatment (T)		0.0001	0.0001	0.0001	0.0001
Y×T		0.0230	0.0001	0.0035	0.0001

^1)^ CP, conventional practice treatment; ICP, improvement of conventional practice treatment; HY, high-yield management treatment; ISM, integrated soil and crop system management treatment. Values followed by different letters within a column in the same year are significantly different at P< 0.05. –, no data.

The effects of treatment, year, and their interaction on NUE, NFP, and WP were significant (**Table 6**). Over 3 years, NUE among the integrated treatments followed the ranking ICP > ISM > HY > CP. The average NUE for ISM was 4.15% lower than that for ICP and was remarkably higher than that for HY and CP by 26.36% and 52.37%, respectively. NFP among the integrated treatments exhibited the same ranking as NUE. The average NFP for ISM was 7.26% lower than that for ICP and was 25.81% and 59.75% higher than that for HY and CP, respectively. Over 2 years, WP among the integrated treatments followed the ranking ISM ≥ HY > ICP > CP. The WP obtained with ISM was significantly higher than that with HY in 2014−2015, whereas no significant difference in WP was observed between ISM and HY in 2013−2014. The WP averaged across 2 years for ISM was 3.63%, 8.28%, and 38.10% higher than that for HY, ICP, and CP, respectively.

### 3.7 Correlation analysis

The correlations between RLD and grain yield, WP, PANU, AGN_M_, and soil water consumption are presented in **Table 7**. WP, PANU, and soil water consumption showed significant positive correlations with RLD in all soil layers. RLD in the 0- to 120-cm soil layers was positively related to grain yield, whereas RLD in the 120- to 140-cm soil layer was not correlated with grain yield. There were no significant correlations between AGN_M_ and RLD in the 80- to 140-cm soil layers, but AGN_M_ and RLD in the 0- to 80-cm soil layers were significantly positively correlated.

**Table 7:** Correlation analysis between root length density (RLD) in different soil layers and grain yield, water productivity (WP), post-anthesis N uptake (PANU), aboveground N uptake at maturity (AGN_M_), and soil water consumption.

	RLD at the depths						
	0−20 cm	20−40 cm	40−60 cm	60−80 cm	80−100 cm	100−120 cm	120−140 cm
Grain yield	0.78**	0.81**	0.93**	0.82**	0.64*	0.59*	0.46
WP	0.88**	0.85**	0.95**	0.90**	0.80**	0.85**	0.77**
PANU	0.84**	0.82**	0.94**	0.86**	0.75**	0.82**	0.74**
AGN_M_	0.68*	0.72**	0.82**	0.72**	0.53	0.32	0.16
Soil water consumption	0.95**	0.84**	0.95**	0.97**	0.91**	0.85**	0.75**

^*^ and ^**^, significant differences at the P< 0.05 and P< 0.01 levels, respectively.

## 4 Discussion

In the present study, integrated management practices showed the combined effects on crop N accumulation at anthesis. AGN_A_ was the highest for HY, followed by ISM, and the lowest for ICP and CP. Previous studies have exhibited that optimized N management (Meng et al., 2013; Hartmann et al., 2015; Lu et al., 2015), proper irrigation (Guo et al., 2014; Wang et al., 2014; Ye et al., 2022), appropriately increasing seeding rate (Dai et al., 2013; Dai et al., 2014) can significantly increase plant N accumulation. However, late sowing may typically reduce N accumulation (Dai et al., 2017; Yin et al., 2018). The abovementioned results indicate that the increased the seeding rate, when combined with the optimized fertilization and irrigation management practices, can compensate for the decrease in N accumulation caused by late sowing. In wheat, N accumulation and redistribution are important processes that determine crop production and grain N accumulation. In general, NR can contribute 50% to 95% of N-grain, and PANU can contribute 5% to 50% (De Ruiter and Brooking, 1994; Kichey et al., 2007). NR is highly dependent on AGN_A_, and PANU is relevant to soil N availability. Both NR and PANU decreased with N deficiency (Bly and Woodward, 2003; Gaju et al., 2014). Moreover, increasing irrigation could reduce NR and increase PANU (Mon et al., 2016). Compared with CP, ICP obtained a higher NR due to its higher AGN_A_, lower N supply and late N application, and lower irrigation amount. Similarly, the higher NR with ISM was mainly contributed to the increased AGN_A_ and the optimized N fertilization and irrigation management. On average, NR contributed 82.86% to 91.38% of N-grain, whereas PANU contributed 8.62% to 17.14%. In comparison to other integrated treatments, wheat grown under ISM supported the highest PANU and CP_PANU_. Thus, the higher NR and the highest PANU were obtained with ISM, which ultimately resulted in the higher N-grain.

To evaluate NUE and N loss precisely, we paid attention to crop-soil system and studied the N balance under integrated management strategies. Crop production alters N balance in AGN_M_, INR, and INL, ultimately affecting NUE and environmental health (Liu et al., 2018; Li et al., 2022). In the present study, ISM supported better N balance by producing a high level of AGN_M_ and decreasing INR_M_ and INL. The AGN_M_ was the highest for HY, followed by ISM, and the lowest for CP. The higher AGN_M_ obtained with ISM was attributed to its higher AGN_A_ and highest PANU. N fertilization both satisfies the needs of wheat growth and is responsible for the agro-environmental pollution due to denitrification and leaching (Fageria and Baligar, 2005; Sainju et al., 2017). Over-irrigation can also lead to high INR in the soil layers (Kang et al., 2017). Significantly higher INR_M_ and INL were observed for CP and HY due to the higher INR_S_ and highest NFA than for ICP and ISM, which results in resource waste and increases the possibility of environmental pollution. The soil N pools cannot expand forever, and, at high levels of N input, saturation values may soon occur. Along with the highest NFA, the lowest AGN_M_ for CP and the highest ANM for HY may be the common influential causes of excess soil N, leading to high levels of N waste under CP and HY. An interesting finding is that INL under all integrated treatments decreased with years, which is mainly attributed to the year-on-year increased AGN_M_ and the slightly decreased N inputs. In addition, the NNI at anthesis can be used as a diagnostic tool for determining crop N status at canopy level (Lemaire and Gastal, 1997). Values more than 1 imply a luxury N consumption, whereas values lower than 1 imply a N deficiency (Lemaire et al., 2008). In our study, the average NNI_A_ value was the highest for HY, followed by ISM, and the lowest for ICP and CP. Moreover, the values of NNI were all more than 1, which implied that wheat grown under all the integrated treatments was in situation of a luxury N consumption. Despite achieving better N balance and higher NUE, there are still great possibilities for improvements in agronomic management practices used under ISM.

With the increase in the total irrigation input, the total water consumption increased. A case study in 2014−2015, the highest total water consumption was obtained with CP and the lowest total water consumption was obtained with ISM. This was mainly due to CP and ISM, respectively, having the highest and the lowest irrigation amount. Compared with HY, the lower total water consumption with ICP was mainly due to its lower soil water consumption. Despite of the lowest irrigation amount, ISM obtained a relatively adequate water supply due to the effective use of soil water storage. In general, the root distributions are generally incorporated into considerations of soil water absorption and grain yield production. Agronomic practices can significantly affect the growth and spatial distribution of root systems. Significant increases in RLD at each soil depth were observed as seeding rate increased (Dai et al., 2014), and delayed sowing resulted in a reduction in RLD (Dai et al., 2017). A positive quadratic relationship between root weight density and amount of N application was observed (Wang et al., 2014). Moreover, proper irrigation can optimize the spatial distribution of roots (Man et al., 2016; Feng et al., 2023) and promote deeper root penetration into the soil (Wang et al., 2014). In the present study, the root distribution in 0- to 140-cm-depth soil was significantly affected by integrated management practices. Owing to further increased seeding rate, appropriate high N application, and proper irrigation input, the largest RLD at anthesis at each soil depth was obtained with ISM and so did the soil water consumption at the corresponding soil depths. This trend reflected that of the total soil water consumption. The growth of root system in the soil layer is crucial for soil water absorption (Guan et al., 2015; Feng et al., 2023). The larger RLD in the soil layers, the more soil water was absorbed by roots to satisfy the needs of plant growth at the late growth stage, which is conducive to achieving the higher grain yield (White et al., 2015). In the present study, the larger and deeper RLD was obtained with ISM, which implied that the ability to capture the deeper soil water and utilization efficiency of stored soil water under ISM was the highest. In addition, the correlation with grain yield was significant; namely, the increases in the deep root systems were conducive to utilization of deep soil water, which may be one of the major reasons for the higher grain yield and WP.

## 5 Conclusions

The integrated management strategy (including appropriately delaying sowing date, increasing seeding rate, and optimizing fertilization and irrigation management) used under ISM could increase grain yield, promote N balance, and improve NUE and WP. The grain yield obtained with ISM was 95.86% of that with HY and was significantly higher compared with ICP and CP across all 3 years. ISM promoted N balance as relatively higher AGN_M_, lower INR_M_, and lowest INL. The average NUE for ISM was 4.15% lower than that for ICP and was significantly higher compared with HY and CP. The NFP exhibited the same trend as NUE. The increased soil water consumption under ISM was mainly due to its increased RLD. ISM improved WP by about 3.63%–38.10% in comparison with other integrated management strategies. Taking high grain yield, better N balance, and high NUE and WP into consideration, ISM can be considered a recommendable management strategy in the target region.

## Data availability statement

The original contributions presented in the study are included in the article/supplementary material. Further inquiries can be directed to the corresponding authors.

## Author contributions

DP and XD designed the experiments, managed the projects and guided the writing of the article. HX, ML, YT, FZ, and WC performed the experiments. HX performed the data analysis and wrote the manuscript. MH gave useful suggestions during the process of this article. All authors contributed to the article and approved the submitted version.
